# Road map to scaling-up: translating operations research study’s results into actions for expanding medical abortion services in rural health facilities in Nepal

**DOI:** 10.1186/1478-4505-12-24

**Published:** 2014-05-13

**Authors:** Mahesh Puri, Shophika Regmi, Anand Tamang, Prabhakar Shrestha

**Affiliations:** 1 Center for Research on Environment Health and Population Activities, Kusunti, P.O. Box 9626, Kathmandu, Nepal

**Keywords:** Auxiliary nurse midwives, Female community health volunteers, Medical abortion, Nepal, Outreach health facilities

## Abstract

**Background:**

Identifying unsafe abortion among the major causes of maternal deaths and respecting the rights to health of women, in 2002, the Nepali parliament liberalized abortion up to 12 weeks of pregnancy on request. However, enhancing women’s awareness on and access to safe and legal abortion services, particularly in rural areas, remains a challenge in Nepal despite a decade of the initiation of safe abortion services.

**Methods:**

Between January 2011 and December 2012, an operations research study was carried out using quasi-experimental design to determine the effectiveness of engaging female community health volunteers, auxiliary nurse midwives, and nurses to provide medical abortion services from outreach health facilities to increase the accessibility and acceptability of women to medical abortion. This paper describes key components of the operations research study, key research findings, and follow-up actions that contributed to create a conducive environment and evidence in scaling up medical abortion services in rural areas of Nepal.

**Results:**

It was found that careful planning and implementation, continuous advocacy, and engagement of key stakeholders, including key government officials, from the planning stage of study is not only crucial for successful completion of the project but also instrumental for translating research results into action and policy change. While challenges remained at different levels, medical abortion services delivered by nurses and auxiliary nurse midwives working at rural outreach health facilities without oversight of physicians was perceived to be accessible, effective, and of good quality by the service providers and the women who received medical abortion services from these rural health facilities.

**Conclusions:**

This research provided further evidence and a road-map for expanding medical abortion services to rural areas by mid-level service providers in minimum clinical settings without the oversight of physicians, thus reducing complications and deaths due to unsafe abortion.

## Background

Nepal, a small country with a population of about 28 million, is predominantly rural (83% of people live in rural areas) [1] and is one of the poorest countries in the world, having a per capita income of about US$ 750 in 2013 [2]. Approximately 70% of women have not received education beyond primary school and only 35% are literate [2]. The maternal mortality ratio in Nepal is still one of the highest in South Asia, although the ratio has dropped from 539 deaths per 100,000 live births [3] to 281 deaths per 100,000 live births [4] between 1996 and 2006.

Unsafe abortion was found to be the third leading direct cause of maternal deaths in Nepal [5]. The identification of unsafe abortion among the major causes of maternal deaths, along with the emergence of national and international women’s rights movements and active coordination of and support from local non-government organizations, civil societies, and public sectors to ensure safe motherhood and rights of women [6], in 14 March 2002 and under the 11^th^ amendment to the Country Code, the Nepali parliament approved legalization of abortion after nearly three decades of the reform efforts [7]. Previous laws did not allow abortion under any circumstances classifying it as an infanticide and many women who had had an abortion were imprisoned [8-10]. Under the new law, abortion is permitted on request up to 12 weeks of pregnancy for any reason, up to 18 weeks of pregnancy in the case of rape or incest, and up to any gestation in case of risk to the woman’s health and life or if the fetus is deformed or incompatible with life [11].

Safe abortion services first started in March 2004 with surgical abortion by manual vacuum aspiration. Second-trimester procedures were introduced in 2007, and senior nurses who had prior training in post-abortion care began providing services in 2008. Manual vacuum aspiration was the only safe abortion technology used until 2009 in Nepal. Recognizing the limitations of surgical abortion service expansion in rural areas with difficult landscape and lack of service providers, and in line with the Safe Abortion Service Directives, 2065 B.S. (2008), the Government of Nepal introduced medical abortion on a pilot basis in six districts through 32 accredited comprehensive abortion care centers for a period of one year in 2009 [12]. The experience gained from the pilot phase of medical abortion services (96.1% success rate) encouraged the Government to scale up medical abortion services in all accredited abortion clinics throughout the 75 districts of the country in a phased manner [13]. However, medical abortion service is limited to a relatively small number of accredited governmental and non-governmental centers, including selected primary health centers and health posts.

Despite achievements in the pilot phase, knowledge and use of medical abortion was very low. The evaluation study of pilot medical abortion services carried out by the Center for Research on Environment Health and Population Activities in 2009, showed that only 15% of the 1,093 married women of reproductive age heard about medical abortion [14]. The Government records from the six medical abortion pilot districts revealed that, during the pilot phase (January-June 2009), of the total women who had an abortion only 6.1% had opted for medical abortion [14].

The evaluation study also pointed out the absence of a full time counselor at the government facilities to enable women to make an informed choice on medical abortion, negative attitudes and low confidence of providers towards medical abortion, a policy that hindered women living more than 2 hours travelling distance from the health facility to use medical abortion, and the requirement for repeat visits of women to the clinic, as some of the major barriers in the utilization of medical abortion services [14,15].

It is widely believed that medical abortion has the potential to expand access of abortion to rural areas which lack physicians and health care infrastructures [16-20]. Nepal is making good progress in expanding medical abortion services; however, enhancing women’s awareness on and access to safe and legal abortion services, particularly in rural areas, remains a challenge despite a decade of the initiation of safe abortion services. Training and mobilizing mid-level health workers, such as auxiliary nurse midwives, in providing safe medical abortion services would be consistent with global trends of task shifting in areas which lack doctors and the infrastructure to provide surgical abortion services [21]. Although medical abortion services by mid-level health workers has been proved effective in increasing access to medical abortion services in Nepal [16] and elsewhere [18-20,22-26], the Nepalese government is reluctant to expand medical abortion services to rural outreach health facilities, particularly in sub-health posts, which are the lowest level of public health facility. This was partly due to the fact that previous studies were conducted in higher level clinical settings where physicians were available to oversee the quality of services. Further, there is no evidence on the provision of medical abortion services by nurses and auxiliary nurse midwives from sub-health post level. Additionally, there is also a lack of adequate evidence on effectiveness of community-based information and referral systems by female community health volunteers to increase medical abortion access and acceptance among rural women. In Nepal, female community health volunteers are highly admired and accepted by rural people. There are currently more than 50,000 female community health volunteers who act as an important source of information for their communities, as a connecting network with government health services, and also as a source of direct health services in a number of areas such as antenatal and postnatal care, HIV/AIDS, childhood illness, and distribution of vitamin A and iron supplementation [27].

To address some of these gaps in evidence, an operations research study was conducted between January 2011 and December 2012 with an aim to determine the effectiveness of engaging female community health volunteers as communication and referral agents and auxiliary nurse midwives to provide medical abortion services from outreach health facilities, particularly from healthpost and sub-health post level in Nepal. This paper describes the main process of implementation of a larger operations research study, key results, and ways of implementing research results for policy advocacy in Nepal. This paper also discusses the challenges and lesson learned from the study.

## Methods

This paper used information from an operations research study that employed a non-equivalent quasi-experimental design with a treatment area (Rupandehi district), where no outreach health facility was providing medical abortion services before this study, and a control area (Kailali district), where medical abortion services were available from outreach health facilities since 2009. In the study, a pre-intervention survey was conducted by interviewing female community health volunteers, physicians, and auxiliary nurse midwives in treatment and control areas. Female community health volunteers, nurses, and auxiliary nurse midwives were then trained and community level interventions were implemented to raise awareness on medical abortion. For the end-line evaluation, an interview with female community health volunteers, nurses, and auxiliary nurse midwives and an exit interview with women who received medical abortion services were conducted. Service statistics maintained by the study sites were also reviewed. Bivariate analysis was performed to assess the observed differences on the knowledge and attitudes of female community health volunteers about medical abortion and performances of auxiliary nurse midwives, before and after the intervention. The protocol and research instruments were approved by the ethical committee of Nepal Health Research Council and Ethical Review Committees of the World Health Organization, Geneva.

## Results

### Setting up the study

Three consecutive meetings with district level stakeholders in the treatment area were held, each chaired by the district public health officer. The first meeting formed a District Support Committee of nine members to provide the necessary guidance and support to the project. Members of the committee were the district public health officer, public health nurse, and representatives from health care institutions (zonal and district hospitals, primary health care center), non-governmental organizations, and the Family Planning Association of Nepal.

Study areas and facilities were also selected during the first meeting. In the treatment area, 8 outreach health facilities: 1 primary health care center, 5 health posts, and 2 sub-health posts; and 126 female community health volunteers from 10 village development committees with 10 female community health volunteers from each ward, were selected. Likewise, 4 outreach health facilities: 2 primary health care centers and 2 health posts; and 96 female community health volunteers from 10 village development committees were selected in the control district. Medical abortion services were available from all 4 outreach health facilities selected for this study in the control area while this was not the case in the treatment area.

Medical abortion service fees for each service seeker was also standardized and it was decided that these should not exceed NRs 500 (US$ 5), and should cover the costs of medicines [NRs 330 (US $ 3.3)], service provider [NRs 100 (US $1)], and health post/sub-health management committee [NRs 70 (US$ 0.7)]. This amount was agreed upon by the District Support Committee.

A variety of information, education, and communication materials, such as brochures, follow-up cards, referral cards, and self-assessment cards, were developed for dissemination in the treatment area. Three hundred packs of medical abortion medicines were supplied to the district public health office. Furthermore, 2 manual vacuum aspiration sets were provided to each health facility and 2,500 pieces of urine pregnancy test kits were provided to female community health volunteers. Three hundred copies of self-assessment cards to be used by service seekers, comprehensive abortion care registers, and record forms were also delivered to the outreach health facilities through the district public health office in the treatment district. In addition, special referral cards were given to female community health volunteers to deliver the card to women with positive test results and either wanting to continue their pregnancy or terminate it.

The program in the treatment area was implemented at two levels – the facility and community levels. At the facility level, a three-day training on medical abortion as per the national medical abortion protocol was provided to 13 skilled birth attendant-trained nurses and auxiliary nurse midwives in coordination with the Family Health Division and National Health Training Centre. Nurses and auxiliary nurse midwives were trained on unbiased counseling, manual vacuum aspiration and medical abortion, minor complication management, and early identification and timely referral of adverse conditions to referral centers. The selected health facilities in the treatment area were certified by the Family Health Division and National Health Training Centre to provide medical abortion services.

Regular monitoring and monthly data collection from the service centers was carried out by a field coordinator based in the treatment area. Similarly, the District Public Health Officer and the Public Health Nurse of the treatment area monitored and provided support in service delivery. In addition, onsite appreciative enquiry and skill enhancement support to the service providers were arranged by two doctors.

At the community level, a two-day training was provided to 120 female community health volunteers to disseminate information about unintended pregnancy prevention, abortion law and rights, safe and legal abortion services, health consequences of unsafe abortion, safety and efficacy of medical abortion, importance of post-abortion contraception, and availability of medical abortion services at health and sub-health post through trained nurse and auxiliary nurse midwives. In addition, they were trained on performing urine pregnancy tests and the use of referral and follow-up cards.

Information on medical abortion service provision in the treatment area was aired through local radio for 4 months and also published in local newspapers. Female community health volunteers organized social events, such as a talk program by senior health providers and opinion leaders, street drama, and quiz competitions. In addition, information on abortion law and availability of medical abortion services were disseminated through Mother’s Groups, outreach clinics, and household visits by female community health volunteers in the treatment area. Furthermore, trained female community health volunteers conducted urine pregnancy tests of women during household visits and referred them to health post, primary health care center, or hospital for comprehensive abortion care or antenatal care, as required. These women were followed-up by female community health volunteers for post-abortion contraceptive use. Periodic monitoring and support at the community level were provided by team members based in the treatment area.

Pre-intervention and post-intervention individual interviews with female community health volunteers and auxiliary nurse midwives were conducted, aimed to examine whether trained auxiliary nurse midwives are able to provide medical abortion services from outreach clinics (health post/sub-health posts) independently and confidently, and whether female community health volunteers can become effective communicators, motivators, and referral agents for medical abortion in rural areas. In addition, data were collected through exit interviews with women who had received medical abortion services in treatment and control areas to examine the differences in perceptions on medical abortion services among these groups. Moreover, 12-month service statistics maintained by the study health facilities were also reviewed. Following are the major findings of the operations research study, while detailed findings of the study have been published elsewhere [28].

### Salient findings

•There was a significant increase in knowledge about abortion law and medical abortion among female community health volunteers in the treatment area compared to the control area. For example, knowledge about the legal conditions for abortion increased from 66% at the pre-intervention survey to 100% at the post-intervention survey in the treatment area while it remained at the same level in the control area. Knowledge about the name of approved medical abortion drugs for early first trimester abortions increased from 1% in pre-intervention to 97% at the post-intervention survey in the treatment area. Similarly, at the pre-intervention survey, about 12% of the female community health volunteers in the intervention group and 15% in the control group mentioned ‘medical abortion is a low risk method’ as one of the advantages of medical abortion services. At the post-intervention survey, these percentages increased by 70% in the intervention group and 39% in the comparison group.

•Female community health volunteers in the treatment area effectively and confidently conducted urine pregnancy tests at household level and confirmed pregnancy, offering advice on medical abortion to those not intending to keep their pregnancy, and persuading them for post-abortion family planning. For example, a total of 584 urine pregnancy tests were performed by the female community health volunteers in the treatment area in the 12-month period. Women who tested positive were either referred to antenatal care services or abortion services depending on their needs and choices.

•At the pre-intervention survey, almost none of the female community health volunteers in the treatment area and 25% of the female community health volunteers in the control area mentioned that they refer women opting for medical abortion to health and sub-health posts. In the post-intervention survey, these percentages increased by 80% in the treatment area and only 5% in the control area.

•Service statistics showed that 307 women in the treatment area had received medical abortion services from auxiliary nurse midwives in the 1-year period. Abortion success rate was 98.4%. There were five incomplete abortions (1.6%), four of these women received manual vacuum aspiration services for incomplete abortion from same providers and one woman was referred to the nearest zonal hospital.

•Exit interviews with 124 women in the treatment area who received medical abortion services from auxiliary nurse midwives at outreach health centers showed that 96% of the women in the treatment area reported that the service of the health facility met their expectations and they would recommend their friends for the services.

•Exit interviews with women in the treatment area also reported that self-assessment or follow-up cards were found to be very useful (98.7%) and effective in developing self-confidence and assessing abortion outcomes among women.

•Interviews with auxiliary nurse midwives in the treatment area revealed that all auxiliary nurse midwives participating in this study were confident about providing medical abortion independently.

•The study led to the accreditation of sub-health posts as medical abortion service sites for the first time in the country and demonstrated that sub-health posts with trained auxiliary nurse midwives and nurses can provide medical abortion services safely and effectively. In addition, the study standardized the fee for medical abortion services in study outreach health facilities.

### Translating research findings into action

It is crucial that study results inform program and policy in country and elsewhere. In order to ensure this, the following activities were undertaken.

#### Dissemination of study findings

Key findings of this study were disseminated at the national stakeholders meeting, chaired by a senior demographer of the Family Health Division, Ministry of Health and Population, Nepal. Participants of the meeting were Ministry of Health and Population (MoHP) officials, representatives from the Ministry of Women, Children and Social Welfare, National Women’s Commissions, and the World Health Organization, the director of the Maternity Hospital, the registrar of the Nursing Council, and representatives from international and local non-governmental organizations. After the discussion on and the appreciation of the evidence generated by this study, participants believed on the importance of task shifting for medical abortion services in the context of a country such as Nepal and were convinced that mid-level providers can independently and effectively provide medical abortion services. The Chairperson of the meeting encouraged replicating and expanding medical abortion up to sub-health post level delivered by nurses and auxiliary nurse midwives, and assured that the required changes will be made in policies and that an enabling environment will be created to expand medical abortion up to low level health service centers such as birthing centers.

#### Policy advocacy at district level

Result-dissemination meetings were organized at the two districts. The first meeting was organized in the treatment area (Rupandehi district), chaired by the District Public Health Officer. Members of the District Support Committee, medical abortion service providers, in-charges of health posts/sub-health posts, chairpersons of health post/sub-health post management committees, and other district level stakeholders, attended the meeting. After sharing key results, the meeting mainly focused on follow-up activities as well as the potential scale-up of medical abortion services in the remaining outreach health facilities of the district. The district public health office formally committed to continue to provide medical abortion services at the study outreach health facilities and expand medical abortion services in other rural areas using the same approach.

The second result-dissemination meeting was organized in Chitwan district with the chairpersonship of the District Public Health Officer of Chitwan. The participants were district level key stakeholders, such as government officials, in-charges of health posts/sub-health posts, auxiliary nurse midwives, safe abortion service providers, and members of Reproductive Health Coordination Committees. The District Public Health Officer and service providers of the treatment district shared their experiences of managing and implementing medical abortion services through outreach health facilities and their confidence in providing the service. All the participants felt the need to replicate medical abortion services through a sub-health post level not only in Chitwan, but also throughout the country. The District Public Health Officer of Chitwan made a public commitment to introduce medical abortion services through nurses and auxiliary nurse midwives from sub-health posts in the district.

#### Advocacy/interactive meetings with the Ministry of Health and Population

Following the national level dissemination, two advocacy meetings with the Family Health Division of the Ministry of Health and Population and the Technical Committee for Implementation of Comprehensive Abortion Care services were organized. Participants of the meeting appreciated the approach used in this study and agreed that expanding medical abortion services through trained nurses in rural health and sub-health posts is safe, effective, and feasible. The meeting also recommended the government to scale up the medical abortion services using the approach demonstrated by this study. Consequently, the Ministry of Health and Population is preparing to scale up this approach in several districts in a phased manner in partnership with various development partners.

## Discussion

This study has demonstrated that careful planning and implementation, continuous advocacy, and engagement of key stakeholders including concerned higher level government officials from the beginning of the project are the key strategies for a successful completion of this study. These factors were not only crucial for effective accomplishment of the study but were also instrumental for translating research results into action and policy change.

Expanding the provider base to include a range of other health workers is a recognized strategy to expand access to health services and scale up implementation of interventions of public health significance. It is recognized as being a critical need for safe abortion care as well. Medical abortion, as an effective and recommended technology, has made it even more relevant to expand health workers’ roles in provision of safe abortion care and to look at self-assessment as a way of reducing the need for health worker time and resources. There is a growing body of research evidence on the issue [29]. Additionally, in several countries, the use of some cadres of non-physician providers to provide safe abortion services is already part of policy and practice [26,30,31]. This study further confirms that medical abortion services can be provided by nurses and auxiliary nurse midwives safely and effectively, not only at the higher level health facilities where physician oversight is available, but also to rural lower level health facilities where such support and oversight are not available. As evidenced by this study, the Ministry of Health and Population and other key partners working in areas of abortion care services in Nepal are expanding medical abortion services by mid-level providers in lowest level of health facilities. This will yield further evidence and build confidence in the policy makers for the scaling-up of this approach throughout the country in the near future.

Despite these achievements, the study faced some challenges at different levels. First, there is no global guideline on mid-level provision of safe abortion care. While the *Safe Abortion: Technical and Policy Guidance for Health Systems of World Health Organization, 2012*, refers to the role of mid-level providers as being appropriate, it does not make upfront specific recommendations about cadres of providers and the specific tasks or methods for which task shifting is appropriate. Second, staff turnover (transfer of auxiliary nurse midwives outside of the study outreach health facility) was challenging to continue to provide services at the study facilities as new untrained auxiliary nurse midwives would not be able and allowed to provide medical abortion services from these health facilities. To avoid the shortage of trained auxiliary nurse midwives in the selected health facilities, additional numbers of nurses and auxiliary nurse midwives were trained during the intervention phase. A third challenge was the resistance of health post and sub-health post in-charges in delegating this task to their juniors – nurses and auxiliary nurse midwives. Health post and sub-health post in-charges are the people who manage out-patient departments in these facilities and refer women seeking abortion services to auxiliary nurse midwives. This was a particular problem at the beginning of the study; however, it was later sorted out and positive support was received from the facilities’ in-charges. Good networking and referral also needs to be done between nurses and in-charges of health posts/sub-health posts within the facility.

## Conclusions

The Nepali government has a policy to expand medical abortion services in rural areas. This study showed that safe abortion services can be provided by trained nurses and auxiliary nurse midwives not only at the higher level health facilities where physician oversight is available, but to rural lower level health facilities where such support and oversight are not. Therefore, it is important for the government to consider accrediting sub-health posts with a trained auxiliary nurse midwife for medical abortion services. Moreover, this study found that female community health volunteers can be instrumental in informing women about medical abortion, conducting urine pregnancy tests, referring women to a safe place for abortion, and providing post-abortion contraceptive counseling. Therefore, they can be used as potential change-agents in creating awareness about medical abortion and referrals in rural areas of Nepal.

## Competing interests

The authors of this paper declare that they have no competing interests.

## Authors’ contributions

MP and AT jointly designed the study, developed study instruments, supervised data collection, prepared an outline of the paper, and reviewed the content of the manuscript. MP and SR analyzed the data and prepared the manuscript. PS was involved in data collection, training of the research team, and interpreted the findings. All authors read and approved the final content of the manuscript.
